# A Neural Device Inspired by Neuronal Oscillatory Activity with Intrinsic Perception and Decision‐Making

**DOI:** 10.1002/advs.202414173

**Published:** 2025-02-04

**Authors:** Congtian Gu, Guoliang Ma, Mengze Zhang, Hu Shen, Liaoyuan Pu, Yanhe Song, Shilong Yan, Dakai Wang, Kaixian Ba, Bin Yu, Zhiwu Han, Luquan Ren

**Affiliations:** ^1^ State Key Laboratory of Crane Technology Yanshan University Qinhuangdao Hebei 066000 China; ^2^ School of Engineering and Informatics University of Sussex Falmer Brighton BN1 9RH United Kingdom; ^3^ Key Laboratory of Bionic Engineering (Ministry of Education) Jilin University Changchun Jilin 130022 China

**Keywords:** baseline shift, bionic sensors, neural devices, neuronal oscillatory activity, power‐frequency electromagnetic field

## Abstract

Bionic neural devices often feature complex structures with multiple interfaces, requiring extensive post‐processing. In this paper, a neural device with intrinsic perception and decision‐making (NDIPD), inspired by neuronal oscillatory activity is introduced. The device utilizes alternating signals generated by coupling the human body with the power‐frequency electromagnetic field as both a signal source and energy source, mimicking neuronal oscillatory activity. The peaks and valleys of the alternating signal are differentially modulated to replicate the baseline shift process in neuronal oscillatory activity. By comparing the amplitude of the peaks and valleys in the NDIPD's electrical output signal, the device achieves intrinsic perception and decision‐making regarding the location of mechanical stimulation. This is accomplished using a single interface, which reduces data transmission, simplifies functionality, and eliminates the need for an external power supply. The NDIPD demonstrates a low‐pressure detection limit (<0.02 N), fast response time (<20 ms), and exceptional stability (>200 000 cycles). It shows great potential for applications such as game control, UAV navigation, and virtual vehicle driving. The innovative energy supply method and sensing mechanism are expected to open new avenues in the development of bionic neural devices.

## Graphical abstract

Inspired by neuronal oscillatory activity, a neural device with intrinsic perception and decision‐making (NDIPD) is proposed. By mimicking neuronal oscillations, it enables perception and decision‐making at five mechanical stimulation points using a single interface. This approach reduces data transmission, simplifies functionality, and eliminates the need for an external power supply.

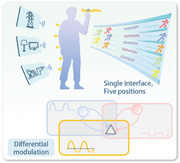

## 1 Introduction

Sensation serves as an objective driver of behavioral decisions in organisms, with the perception and nervous systems playing critical roles as information processing centers and signal carriers. Biologically, the peripheral nervous system, which is responsible for sensation, converts stimulation (e.g., force, light, or sound) into electrical signals that are recognized and processed by the central nervous system. In the era of advanced industries and multidisciplinary fields such as artificial intelligence,^[^ ^1^, ^2^ ^]^ robotics,^[^ ^3^, ^4^, ^5^ ^]^ virtual reality (VR),^[^ ^6^, ^7^ ^]^ and intelligent perception technology,^[^ ^8^, ^9^, ^10^ ^]^ there is a growing demand for real‐time data processing, enhanced perception capability, and efficient decision‐making. However, traditional von Neumann computing systems, which rely on centralized and sequential operations,^[^ ^11^ ^]^ face challenges in handling the bottlenecks caused by exponential increases in data volumes and node numbers. Neuromorphic systems, inspired by the human brain, offer efficient processing capabilities, scalability, and low power consumption. These characteristics position them as a promising solution to overcome the limitations of the von Neumann bottleneck,^[^ ^12^ ^]^ garnering significant attention across various domains.

Numerous studies have been conducted on devices and methods that simulate neuronal perception and decision‐making.^[^ ^13^, ^14^, ^15^, ^16^, ^17^, ^18^, ^19^, ^20^ ^]^ Traditional bionic neural devices rely on discrete sensors to mimic the perception of mechanical stimulation but lack the ability to generate oscillatory signals.^[^ ^15^ ^]^ To address this limitation, researchers have integrated discrete sensing arrays with NbOx‐based memristors, successfully transforming mechanical stimulation into oscillatory signals resembling neuronal activity.^[^ ^16^ ^]^ However, this approach suffers from high energy consumption. Analog neurons based on diffusion memristors have shown potential for reducing energy consumption, but they remain prone to cumulative errors.^[^ ^17^ ^]^ While analog neurons utilizing ring oscillators^[^ ^11^ ^]^ mitigate cumulative errors, they are more expensive and complex to fabricate. Current bionic neural devices are often intricate in structure, involve multiple interfaces, and require external energy input. These limitations hinder their ability to achieve self‐powered operation and align effectively with distributed neuromorphic systems. The challenge, therefore, lies in developing a bionic neural device that features a simple structure, operates with a single interface, requires no external power supply, and can autonomously generate oscillatory signals while achieving intrinsic perception and decision‐making capabilities.

To achieve self‐powered devices, various energy harvesting technologies are commonly utilized, including triboelectric nanogenerators,^[^ ^21^, ^22^, ^23^ ^]^ piezoelectric nanogenerators,^[^ ^24^, ^25^, ^26^ ^]^ and electromagnetic generators.^[^ ^27^, ^28^, ^29^ ^]^ These technologies harness energy from the environment or motion and are characterized by their simple structure and stable output.^[^ ^30^ ^]^ However, their reliance on mechanical motion^[^ ^31^, ^32^, ^33^ ^]^ and the need for cumbersome signal post‐processing.^[^ ^30^, ^31^, ^33^ ^]^ Fortunately, advancements in body‐coupled energy utilization from power‐frequency electromagnetic fields^[^ ^34^, ^35^ ^]^ have opened new avenues for developing self‐powered bionic neural devices that do not depend on mechanical motion. Power‐frequency electromagnetic fields, generated by power cables, industrial machinery, household appliances, and urban traffic, are pervasive in modern environments. The human body, with its large surface area and good electrical conductivity,^[^ ^36^ ^]^ is well‐coupled to these low‐frequency electric fields. As a result, the skin surface can sense alternating oscillating signals, which can serve as a signal source for bionic neural devices. This approach provides a promising solution for mimicking neuronal oscillatory activity in self‐powered systems.

This paper proposes a neural device with intrinsic perception and decision‐making (NDIPD) that uses a power‐frequency electromagnetic field as a signal source, inspired by the baseline shift process in neuronal oscillatory activity. The device generates an alternating signal through the coupling of the human body and the power‐frequency electromagnetic field. This signal serves as both a signal source and an energy provider to simulate neuronal oscillatory activity, eliminating the need for complex oscillatory circuits and external power supplies. By mimicking the baseline shift process in neuronal oscillations, the peak and valley values of the alternating signal are differentially modulated. Intrinsic perception and decision‐making regarding the location of mechanical stimuli are achieved via a single interface. When subjected to spatiotemporally dynamic mechanical stimuli, NDIPD generates a voltage response with asymmetric peaks and valleys. The peak‐to‐valley amplitude ratio exhibits significant regional differentiation, demonstrating strong robustness and stable operation in spatiotemporal discrimination across different environments. This paper first analyzes the working principle of the NDIPD in perceiving and recognizing external mechanical stimulation, followed by decision‐making, based on the processes of perception, transmission, and computation of mechanical stimulation in organisms. Then a performance test platform was developed to assess the effects of various factors, including resistance values, individual differences, mechanical stimulation locations, frequencies, and environmental conditions, on the NDIPD's performance. Finally, the feasibility of the device was demonstrated in applications such as game control, UAV navigation, and virtual vehicle driving. This work advances bionic perceptual neural devices, opening new pathways for the development of efficient parallel neuromorphic systems and facilitating the seamless integration of general artificial intelligence with the metaverse.

## 2 Results and Discussion

### 2.1 Bio‐Inspiration and Design of the NDIPD

**Figure 1:**
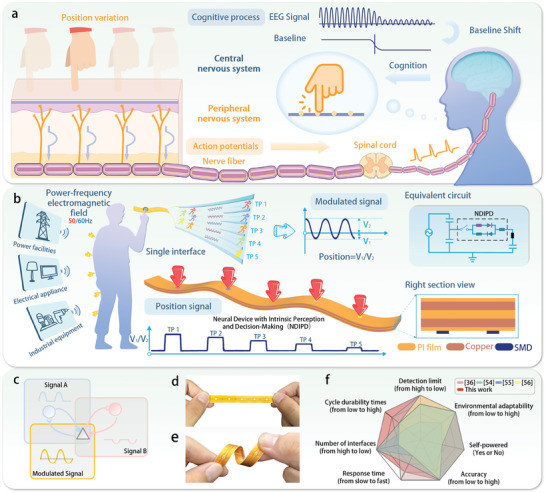
a) The process of biological perception and recognition of external mechanical stimulation. b) The structure and working principle of the NDIPD. c) Schematic diagram of the two branch signals in the circuit of the NDIPD and the voltage response signal generated by their superposition. d,e) Optical images of NDIPD in the straightened and bent states. f) Radar plots comparing the data of this study with those reported in the relevant literature.^[^ ^36^, ^54^, ^55^, ^56^ ^]^

The process of biological perception and recognition of external mechanical stimulation is depicted in **Figure** **1**a. External mechanical stimulations are first converted into receptor potentials by mechanoreceptors in the skin. These receptor potentials are then transmitted to the cerebral cortex via nerve fibers, the spinal cord, and other peripheral nerve postulates.^[^ ^37^, ^38^, ^39^, ^40^ ^]^ Ultimately, the information is processed by a complex and extensive neural network comprising hundreds of millions of neurons, enabling the recognition and perception of mechanical stimulation.^[^ ^41^, ^42^ ^]^ To better understand the intricate relationship between neuronal oscillatory activity and brain information processing, researchers have established links between human perception and neuronal activity using electroencephalograms.^[^ ^43^ ^]^ Studies have shown that neuronal oscillatory activity in the brain exhibits a non‐sinusoidal characteristic, referred to as “amplitude asymmetry” or “baseline shift,” within the alpha and beta frequency bands.^[^ ^43^, ^44^, ^45^, ^46^ ^]^ This phenomenon arises from synchronous asymmetric fluctuations caused by differential modulation of signal peaks and valleys. These fluctuations enhance the brain's ability to integrate information, optimize cognitive functions, and improve decision‐making processes. They are closely associated with perception‐related activities such as action monitoring,^[^ ^47^ ^]^ language comprehension,^[^ ^48^, ^49^ ^]^ response preparation,^[^ ^50^ ^]^ and novelty detection.^[^ ^51^ ^]^ Inspired by this mechanism, a bionic neural device must incorporate three fundamental functions: intrinsic perception, recognition, and decision‐making. In our design, the alternating electric signal generated by coupling the human body with a power‐frequency electromagnetic field serves as the NDIPD's signal source, mimicking neuronal oscillatory activity. The device achieves intrinsic perception, recognition, and decision‐making regarding the position of mechanical stimulation through differential modulation of signal peaks and valleys, effectively simulating the baseline shift process in neuronal activity.

The structure and operational principle of the NDIPD are illustrated in Figure 1b. Various electrical devices, such as power facilities, household appliances, and industrial equipment, produce electric field effects during operation. These effects subsequently generate electric fields in the surrounding environment.^[^ ^52^ ^]^ These fields, primarily at power frequency (50/60 Hz), dominate the surrounding environment.^[^ ^35^ ^]^ When exposed to this power‐frequency electromagnetic field, weak alternating voltage signals are induced at the fingertips due to the coupling interaction between the electric field and the human body. Upon contact with a surface, these signals are transmitted through the fingertip skin and coupled into the NDIPD. The NDIPD comprises four main components: the substrate layer, conductive layer, cover layer, and component layer. The substrate layer, made of polyimide (PI), offers excellent flexibility and heat resistance^[^ ^53^ ^]^. Copper plating on both sides of the substrate forms the conductive layers, which are patterned to construct the circuit structure. An insulating film coats the conductive layer's outer surface, providing insulation and environmental protection. The component layer, located at the base, facilitates circuit construction. The device features five touch points (TP1 to TP5) on its surface, each corresponding to a distinct circuit structure that generates a unique voltage response signal. Figures S1–S4 and Note S1 (Supporting Information) detail the working principle and structure of the NDIPD. When a specific touch point is contacted, the distribution of series resistances in the circuit branches is altered. The two branches pass through diodes oriented oppositely, producing a signal A containing only the positive half‐wave and a signal B containing only the negative half‐wave (Figure 1c). The superimposition of these signals forms a voltage response signal with distinctive peak‐to‐valley characteristics. The touch position is identified by analyzing the ratio of the peak amplitude (V2) to the valley amplitude (V1), a metric resistant to external factors (Note S1, Supporting Information). Figure 1d,e display optical images of the NDIPD, highlighting its excellent flexibility, bending performance (Figure S5, Supporting Information), and lightweight characteristics (Figure S6, Supporting Information), attributed to its reduced thickness. Figure 1f compares the NDIPD's performance with existing devices, showcasing its advantages in detection limit (Figure S7, Supporting Information), environmental adaptability, self‐powered capability, accuracy (Figure S8, Supporting Information), response time, number of interfaces, and cycle durability times (Figures S9 and S10, Supporting Information), specific data are shown in Table S1 (Supporting Information). These attributes make the NDIPD a highly effective and robust bionic neural device.

### 2.2 Performance and Characterization of NDIPD

**Figure 2:**
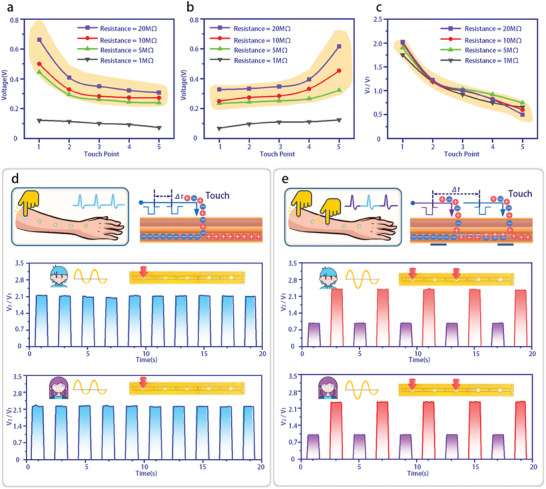
a–c) The peak amplitude V2, the valley amplitude V1, and the peak‐to‐valley amplitude ratio of the voltage response of NDIPD with different resistances when each touch point is subjected to mechanical stimulation. d) Schematic diagram of stimulation applied to TP1 (top left), equivalent charge movement (top right), and the ratio of V2 to V1 under stimulation by volunteer 1 (middle) and volunteer 2 (below). e) Schematic diagram of alternating stimulation applied to TP1 and TP3 (top left), equivalent charge movement (top right), and the ratio of V2 to V1 under stimulation by volunteer 1 (middle) and volunteer 2 (below).

To investigate the impact of different resistance values on the output performance of the NDIPD, the voltage response peak amplitude (V2), valley amplitude (V1), and the peak‐to‐valley amplitude ratio are evaluated for individual resistances of 1, 5, 10, and 20 MΩ. The experimental results are shown in **Figure** **2**a–c. The data reveal that both the peak amplitude (V2) and the valley amplitude (V1) increase as the resistance value rises at the same touch position. This can be explained using Equation S1 (Note S1, Supporting Information). As the total resistance of the NDIPD increases, it accounts for a larger proportion of the voltage in the overall body‐coupled circuit. When the touch position shifts from left to right, the peak amplitude V2 gradually decreases, while the valley amplitude V1 increases. This behavior results from the sequential change in the number of resistors in the two branches as the touch position moves. Following signal clipping by diodes and their superimposition, the voltage response shows a monotonic variation in both the peak and valley amplitudes. The peak‐to‐valley amplitude ratio exhibits distinct regional variations depending on the touch position and resistance values, enabling the NDIPD to accurately identify all touch positions.

In the neurocognitive system, the spatio‐temporal coherence of collective neuronal responses is a fundamental basis for learning. It refers to the consistent responses generated by groups of neurons under brief intermittent stimulation. Similarly, we can examine the response characteristics of the NDIPD using short‐interval stimulation. When external mechanical stimulation is applied to TP3 of the NDIPD at 1 s intervals, positive and negative charges flow to opposite ends under the influence of the diodes (Figure 2d, top). The differing numbers of resistors in the two branches result in variations in the peak and valley of the voltage response signals. After multiple consecutive stimulations, the peak‐to‐valley amplitude ratio of the voltage response signal shows consistent fluctuations (Figure 2d, middle). The application and subsequent withdrawal of mechanical stimulation lead to the rapid generation or loss of signals, resembling processes observed in cognitive functions. Furthermore, due to the differential modulation of neuronal oscillatory activity, the relationship between the peak‐to‐valley amplitude ratio and the stimulation position remains largely unchanged across different volunteers (Figure 2d, below).

The diverse mechanoreceptors, when stimulated in a spatio‐temporal dynamic manner, are activated and transmit signals to the intricate neural networks within the brain, generating differential and consistently repetitive neuronal activity. The peak‐to‐valley amplitude ratio in the voltage response of the NDIPD to mechanical stimulation at different positions shows highly region‐specific characteristics, making the NDIPD an ideal candidate as a brain‐inspired sensing device. It can mimic the spatio‐temporal dynamics of neural network logic, enabling internal perception and behavioral decision‐making. The application of brief mechanical stimulation to TP1 and TP3 at 1 s intervals (Figure 2e, top) leads to the rapid and stable generation of a peak‐to‐valley amplitude ratio with distinct regional differentiation in NDIPD (Figure 2e, middle). Similarly, the relationship between the amplitude ratio and the stimulation position remains largely unchanged when the device is stimulated by different volunteers (Figure 2e, below). The results from multiple evaluations of mechanical stimulation at various positions by different volunteers demonstrate the robust performance of the NDIPD.

**Figure 3:**
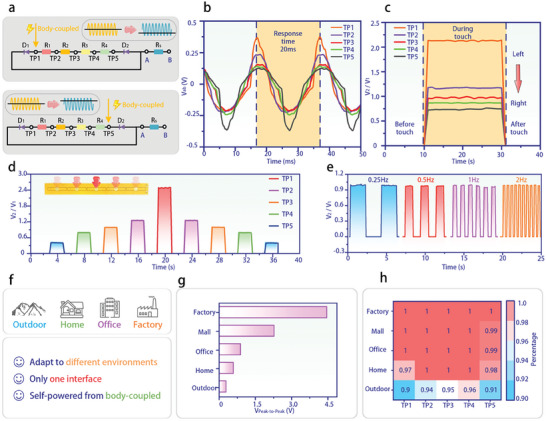
a) Equivalent circuit diagram of the NDIPD when TP1 and TP5 are subjected to mechanical stimulation. b) The signal of the voltage response of each point of the NDIPD when they are subjected to mechanical stimulation. c) the peak‐to‐valley amplitude ratio of the voltage response when each point of the NDIPD is subjected to mechanical stimulation. d) The amplitude ratio of the voltage response when mechanical stimulation is applied sequentially to each point of the NDIPD. e) The amplitude ratio of the voltage response of TP3 when subjected to mechanical stimulation of different frequencies. f) The application scenarios and advantages of the NDIPD. g) Maximum voltage generated by NDIPD in different scenarios (peak‐to‐peak). h) Heat map of response accuracy at each point of NDIPD for different scenarios.

**Figure** **3**a illustrates the equivalent circuit diagrams of the NDIPD in response to mechanical stimulation at TP1 and TP5. The alternating signal produced by the coupling of the human body to the power‐frequency electromagnetic field can be approximated as a constant alternating voltage signal. The two branches of the touch points are represented as multiple resistors and diodes connected in series. When TP1 is subjected to mechanical stimulation, alternating signals are input from the fingertip skin into TP1, forming a conductive path. The signal then splits into two distinct paths. One path bypasses the resistors and passes through diode D1, retaining only the positive half‐wave signal. The other path is amplitude‐modulated by four series resistors (R1‐R4) before passing through diode D2, retaining only the negative half‐wave signal. The two signals merge at Rs. Since both signals have the same phase and frequency but different amplitudes, their superposition results in a signal that is approximately sinusoidal, but with a positive half‐wave amplitude greater than the negative half‐wave amplitude (see Figure 3a above). Similarly, when TP5 is stimulated, the modulation in the two branches is reversed compared to TP1. One signal is amplitude‐modulated by the four series resistors R1–R4 passes through diode D1 and retains only the positive half‐wave signal. In contrast, the other signal bypasses the resistors and passes through diode D2, retention only the negative half‐wave signal. These signals are then superimposed to generate a signal that is approximately sinusoidal, but with the negative half‐wave amplitude exceeding the positive half‐wave amplitude (see Figure 3a below).

To gain deeper insight into the signal characteristics of the NDIPD, we analyze the voltage responses at different touch points. Stimulations at each point generate asymmetrical signals with distinct modulation characteristics. The response time is less than 20 ms (Figure 3b), ensuring synchronization with perception. By extracting the peak and valley amplitudes of the response signals at each point and processing the data, it is evident that the peak‐to‐valley amplitude ratio exhibits clear regional differentiation (Figure 3c). When different mechanoreceptors are subjected to continuous random position stimulation, the brain generates distinct and rapid neuronal activity in response. For instance, when five adjacent points on a person's arm are touched in rapid succession, the brain perceives each point immediately and makes corresponding decisions. To further study the continuous spatio‐temporal response capability of the NDIPD, a test is conducted in which cyclic mechanical stimulation is applied sequentially from TP5 to TP1 and back to TP5 at 1 s intervals (Figure 3d). The NDIPD accurately and rapidly outputs the voltage response signals corresponding to each point.

The response characteristics of mechanoreceptors vary when subjected to mechanical stimulation at different frequencies. For example, Merkel disks are attuned to low‐frequency vibrations, while Meissner's corpuscles are more responsive to high‐frequency vibrations. The nervous system processes these different frequencies through integration and adaptation, ultimately producing positional cognition with frequency‐responsive coherence and vibrational cognition with frequency differentiation. Similarly, the response properties of the NDIPD can be examined by applying stimulation at varying frequencies. Dynamic mechanical stimulation is applied at frequencies of 0.25, 0.5, 1, and 2 Hz to TP3 of the NDIPD (Figure 3e). The results show that the NDIPD exhibits a continuous and stable dynamic voltage response to changes in mechanical stimulation frequency, demonstrating good frequency response consistency.

This indicates that the NDIPD can accurately and continuously respond to a wide range of mechanical stimulation encountered in daily life. The NDIPD is adaptable to various scenarios, offering the advantages of a single interface and the elimination of the need for an external power supply (Figure 3f). To verify the benefits of the NDIPD, five typical application scenarios (factory, mall, office, home, and outdoor) are tested. All tests are conducted with a single interface connection and no external power input.

The diverse range of electrical appliances, in terms of typology, number, and distribution, leads to considerable variations in the magnitude of the field strength. The maximum output voltage of the power‐frequency electromagnetic field from body‐coupled input to the NDIPD also varies, with the order of magnitude as follows: factory > mall > office > home > outdoor (Figure 3g). The proportional modulation characteristics of the NDIPD ensure that the peak‐to‐valley amplitude ratio of the voltage response generated at the same point across different environments remains nearly constant, regardless of the amplitude of the original input signal. This means that the response is solely dependent on the position of the finger touching, independent of the environmental electric field strength. As a result, the NDIPD demonstrates high accuracy across a variety of scenarios (Figure 3h). These findings highlight the NDIPD's exceptional environmental adaptability, waterproof properties (Figure S11, Supporting Information), and user convenience, offering a robust solution for the development of energy‐efficient parallel interactive systems.

### 2.3 Practical Applications of NDIPD

**Figure 4:**
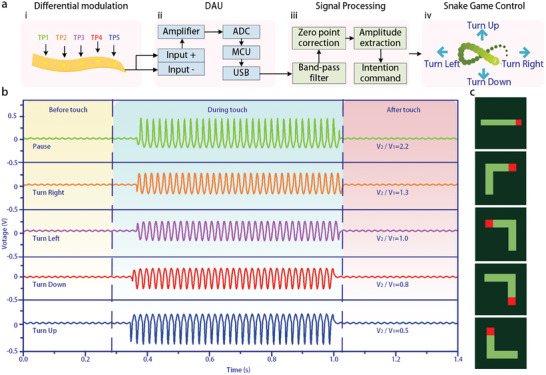
a) NDIPD‐based control of video games. i) The differential modulation mechanism of NDIPD. ii) Data acquisition unit (DAU). iii) Signal processing unit. iv) Control of different movements in the snake game. b) Control signals generated by the NDIPD when subjected to mechanical stimulation and corresponding movement states in the snake game. c) Schematic diagram of a snake executing pause, turn right, turn left, turn down, and turn up control commands in sequence.

As proof of the concept, the NDIPD was used as a neuromorphic interaction control interface for manipulating video games (**Figure** **4**). Upon contact between a finger and the touch point of the NDIPD, a voltage response signal is generated almost instantly. As shown in Figure 4a, the voltage response signal is transmitted through a single interface to a data acquisition unit (DAU), where an operational amplifier amplifies the signal, enabling an analog‐to‐digital converter (ADC) to convert it into a digital signal. After processing by the microcontroller unit (MCU), the signal is transferred via USB to a PC for signal processing and analysis. This includes band‐pass filtering, zero point correction, and feature extraction to facilitate manipulation of the snake game—controlling up, down, left, right, and pause according to pre‐defined commands in the application. The NDIPD consists of five touch points, from left to right, TP1, TP2, TP3, TP4 and TP5, which correspond to the up, down, left, right and pause actions in the snake game.

In the absence of a touch input, the output remains minimal, and the system enters standby mode due to the lack of a loop generated by the NDIPD (Figure 4b). Upon contact between a finger and a valid touch point of the NDIPD, a corresponding voltage response signal is generated (Figure 4b). The peak‐to‐valley amplitude ratio of the voltage response varies with different touch positions. The PC can then manipulate the movement of the snake in the game by recognizing these amplitude ratios (Figure 4c). When the finger leaves the touch point, the voltage response signals dissipate rapidly and return to their initial state, allowing for the transmission of new control commands. Video S1 (Supporting Information) demonstrates the NDIPD as a neuromorphic interaction control interface for video game control. It is clear that the different touch points of the NDIPD enable intuitive control of the snake's movements in the game.

**Figure 5:**
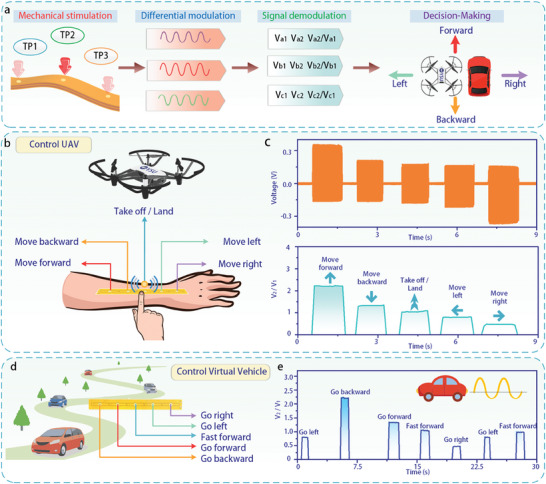
a) Schematic diagram of NDIPD controlling the movement of a UAV or virtual vehicle in each direction. b) Schematic diagram of the control interface and scenarios of the NDIPD‐based interactive control system for neuromorphic UAV. c) Typical NDIPD voltage response signals corresponding to different commands (top) and the peak‐to‐valley amplitude ratio (below). d) Schematic diagram of the control interface and scenarios of the NDIPD‐based virtual vehicle interaction control system. e) The peak‐to‐valley amplitude ratio of the NDIPD voltage response corresponding to different commands.

Accurate perception of intentions relies on the continuous and complete construction of actions, as this process provides correlation and time‐series information between actions, leading to a more precise and consistent representation of behavior. The perception of discrete signals can be challenging for systems attempting to discern the user's true intent. In contrast, interpreting continuous action sequences offers a more nuanced understanding of the user's behavior patterns and dynamic changes, thereby enhancing the system's ability to recognize actions accurately. As a vehicle for neuromorphic interactions, the NDIPD facilitates the construction of continuous response features under static stimulation, distinguishing it from perceptual processes that rely on mechanical motion. To assess the responsiveness of NDIPD to static stimulation, a neuromorphic UAV interaction control system based on NDIPD is proposed. The UAV's take‐off, landing, and movement are controlled using five static touch behaviors, which serve as recognition targets. The control flow of the system is illustrated in **Figure** **5**a, where a continuously differentially modulated signal is generated when a touch point receives static stimulation. After demodulation, the direction of the UAV or virtual vehicle is controlled. The five touch points of the NDIPD, from left to right, correspond to the UAV's movement: forward, backward, take‐off/land, left, and right, respectively (Figure 5b). When a finger is placed on or removed from a touch point on the NDIPD, a continuous signal is generated and then disappears (Figure 5c, top). The voltage response signals at each touch point are demodulated (Figure 5c, below) and transmitted to the UAV, which then performs the corresponding action. Video S2 and Figure S12 (Supporting Information) demonstrate the implementation of a neuromorphic UAV interaction control system using the NDIPD. It is clear that the UAV can be easily controlled through the NDIPD.

The current sensing systems in human‐computer interaction platforms, which act as perceptual bridges between VR, AR, and the metaverse, still face significant limitations, including instability, complexity, and a multitude of interfaces. To enable the implementation of multiple functional commands, it is often necessary to use a sensing array composed of numerous sensing elements. However, this can lead to complicated line interconnections, which may cause instability during interaction and increase the complexity of back‐end signal processing. The NDIPD's excellent single‐interface connectivity and simple construction make it ideally suited for use in a digital interaction environment. To assess the viability of NDIPD in such an environment, this paper presents the design and implementation of a virtual vehicle control platform based on NDIPD. The five touch points are mapped to five virtual vehicle control commands: go forward, go backward, fast forward, go left, and go right (Figure 5d).

When the finger is removed from the touch point of the NDIPD, the voltage response signal rapidly dissipates and returns to its original state. However, the virtual vehicle continues executing the previous command until a new control command is generated. Video S3 (Supporting Information), Figure 5e, and Figure S13 (Supporting Information) demonstrate the process of controlling the virtual vehicle using the NDIPD‐based virtual vehicle control platform. It is clear that by touching different positions on the NDIPD, the virtual vehicle can be easily controlled to perform corresponding actions.

## 3 Conclusion

This paper introduces and designs an NDIPD, developed by differentially modulating the alternating electrical signals generated through the coupling of the human body with a power‐frequency electromagnetic field, mimicking the baseline shift observed in neuronal oscillatory activity. It is demonstrated that by coupling the power‐frequency electromagnetic field, the human body, the NDIPD, and a data acquisition unit, the NDIPD achieves intrinsic perception and decision‐making regarding the position of the mechanical stimulation under spatiotemporal dynamic mechanical inputs. This functionality is accomplished using a single interface, demonstrating exceptional robustness across various environments. The NDIPD exhibits a rapid response time of 20 ms, highlighting its significant potential for application in neuromorphic perception and the development of next‐generation neuromorphic computation systems. Moreover, the NDIPD has been successfully applied in game control, UAV navigation, and virtual vehicle driving, validating its practicality and feasibility as a neuromorphic interaction control interface. The innovative energy supply and sensing mechanism proposed in this study represents a major advancement in bionic perceptual neural devices. This approach is expected to drive technological progress across multiple disciplines, particularly in the development of efficient parallel neuromorphic computation systems and the seamless integration of general artificial intelligence with the metaverse. These contributions offer considerable potential for future applications in diverse fields.

## 4 Experimental Section

### Materials and Fabrication of Devices

The process began by cutting the PI film (25 µm thickness, Shenzhen Haochangsheng Electronics Co., Ltd.), which was coated with a 35 µm copper layer on both sides, into the required shape. The PI film was then affixed with a pad, and positioning and through‐holes were processed using a drilling machine. Once the surface of the PI film had been treated to remove impurities, it was cleaned with a 5% citric acid solution in an ultrasonic cleaner for 5 min to remove the oxide layer and enhance the roughness of the copper surface, thereby improving adhesion between the dry film photoresist and the copper. Next, a dry film photoresist (38 µm thickness, Shenzhen Jiashitong Technology Co., Ltd.) was applied to both copper surfaces. Any air bubbles between the dry film photoresist and the copper layer were removed using a hot press. The printed film was then aligned with the positioning holes on the PI film, ensuring that any remaining air bubbles had been eliminated and that the two film patterns were aligned with the corresponding copper surfaces. The electrode structure of the specimen was sensitized by applying UV radiation for 1 min on each side. Afterward, the specimen was placed in a developer solution (Jinhua Longkexin Technology Co., Ltd.) and removed once the structure was fully exposed. The electrode was placed in a deionized water (Jinhua Longkexin Technology Co., Ltd., weight ratio 1:3) bath containing the etchant. Once the desired electrode structure was achieved, the electrode was promptly removed and rinsed with deionized water. Subsequently, the uncoated PI film (Shenzhen Haochangsheng Electronics Co., Ltd.) was windowed using a laser engraver, with the laser paths automatically generated to align with the specified AutoCAD patterns on both the top and bottom surfaces. Once the three positioning holes were aligned, the layers were pressed using a heat press. Finally, electrically conductive adhesives (YC‐02, Nanjing Xilite Adhesive Co., Ltd.) were used to fill the vias, establishing a connection between the two layers of circuitry. Surface‐mount devices (SMD) were then affixed to the specimen by soldering.

### Signal Processing and Analysis

The data acquisition device (USB‐6001, NI) used in this study collects the voltage response signals from the NDIPD, with real‐time data acquisition implemented through LabVIEW (Figure S14, Supporting Information). In LabVIEW, the amplitude of the peaks and valleys was initially determined using the peak‐finding function. If the total input voltage exceeded a specified threshold, the input was identified as a touch input, and the peak‐to‐valley amplitude ratio was then output. In all other cases, the system remained in an untouched state and produced a value of 0. Finally, the software communicated via UDP packets.

### Characterization and Measurement

A displacement loading platform driven by a stepper motor (57HB56L4‐30DB, Beijing Haijiejiachuang Co., Ltd.) was used to apply mechanical stimulation at varying frequencies. A force gauge (DY920, Freud) was employed to determine the lowest detection limit of the NDIPD, while a data acquisition device (USB‐6001, NI) collected the voltage response signals from the NDIPD (Figure S15, Supporting Information). The application characterization program was developed in Python or Matlab/Simulink, with LabVIEW used for real‐time data acquisition.

## Conflict of Interest

The authors declare no conflict of interest.

## Supporting information



Supporting Information

Supplemental Video 1

Supplemental Video 2

Supplemental Video 3

## Data Availability

The data that support the findings of this study are available from the corresponding author upon reasonable request.
