# Supplementary material for: A Neural Device Inspired by Neuronal Oscillatory Activity with Intrinsic Perception and Decision‐Making
Source: Adv Sci (Weinh). 2025 Feb 4;12(12):2414173. doi: 10.1002/advs.202414173 (PMC11948023; doi:10.1002/advs.202414173)
Supplement: Supplementary file 1 — Supporting Information [file ADVS-12-2414173-s004.docx]

Supporting Information

A Neural Device Inspired by Neuronal Oscillatory Activity with Intrinsic Perception and Decision-Making

Congtian Gu†, Guoliang Ma*†, Mengze Zhang, Hu Shen, Liaoyuan Pu, Yanhe Song, Shilong Yan, Dakai Wang, Kaixian Ba*, Bin Yu*, Zhiwu Han, Luquan Ren

C. Gu, G. Ma, M. Zhang, H. Shen, L. Pu, Y. Song, S. Yan, K. Ba, B. Yu

State Key Laboratory of Crane Technology

Yanshan University

Qinhuangdao, Hebei 066000, China

E-mail: magl@ysu.edu.cn (G. Ma), bkx@ysu.edu.cn (K.Ba), yb@ysu.edu.cn (B.Yu)

D. Wang, G. Ma, Z. Han, L. Ren

Key Laboratory of Bionic Engineering (Ministry of Education)

Jilin University

Changchun, Jilin 130022, China

C. Gu

School of Engineering and Informatics

University of Sussex

Falmer, Brighton BN1 9RH, United Kingdom

† These authors contributed equally to this work.

1. Supplementary Figures


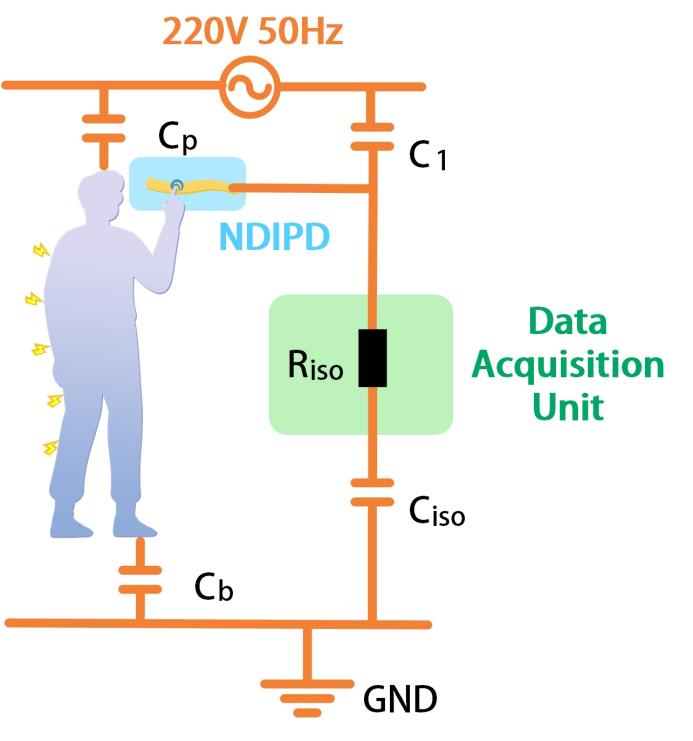


**Figure S1.** Schematic diagram of the coupling effect of the power-frequency electromagnetic field, human body, NDIPD and data acquisition unit.


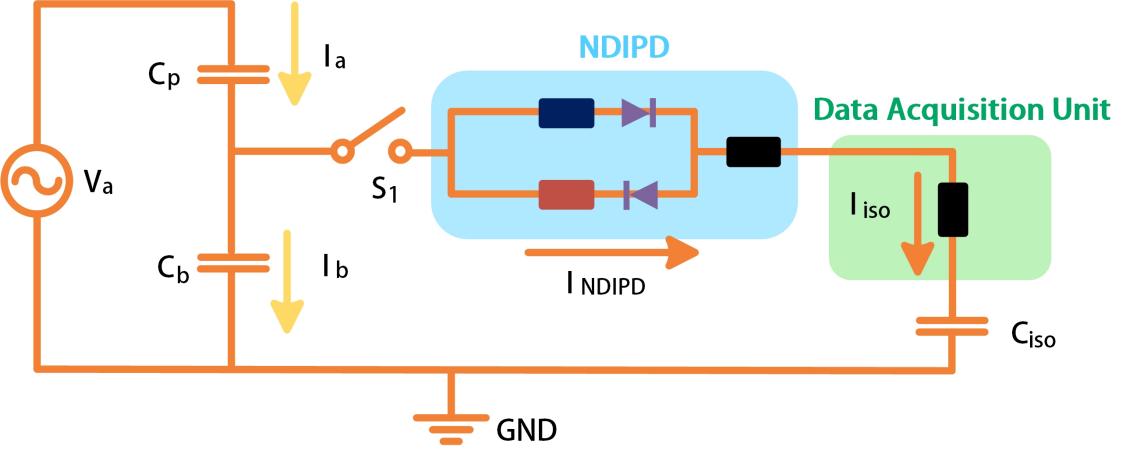


**Figure S2.** Schematic diagram of the working principle of NDIPD.


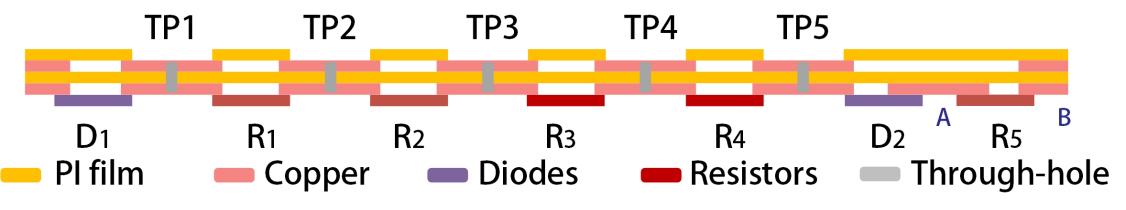


**Figure S3.** Sectional structure of NDIPD.


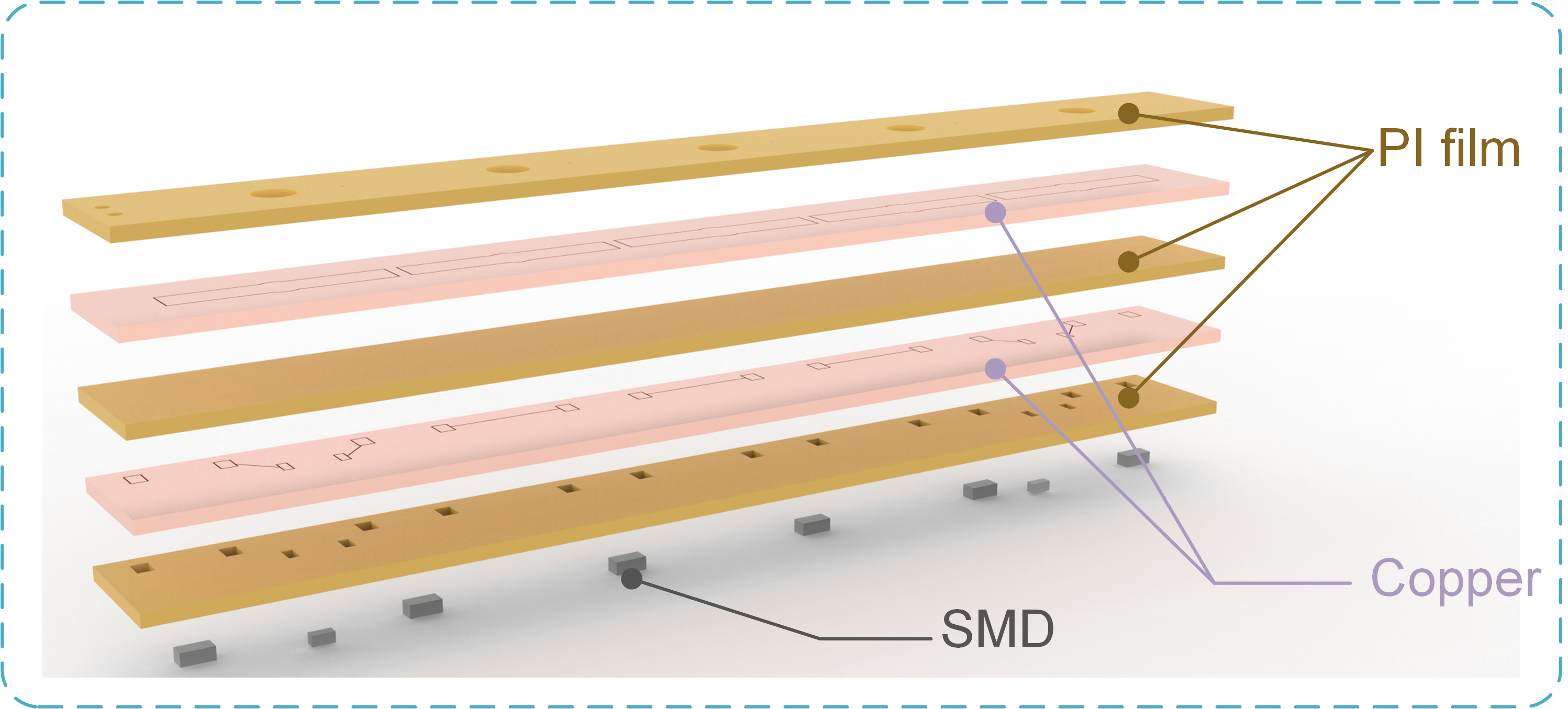


**Figure S4.** NDIPD Schematic diagram of three-dimensional structure.

The basic structure of NDIPD consists of three main components: PI film, conductive copper foil, and surface-mounted components. The top surface of the PI film features windows at touch points, allowing direct finger contact with the upper conductive copper foil. The middle PI film acts as a substrate, offering insulation, flexible support, and firm bonding between the upper and lower conductive copper foils. The lower conductive copper foil is patterned for electrical connections and connected to the upper foil through vias, ensuring seamless transmission of human coupling signals. The lower PI film shields the lower conductive copper foil from external environmental factors, such as oxidation or mechanical damage. Surface-mounted components, such as resistors and diodes, are soldered onto the exposed pads of the lower conductive copper foil to modulate and process signals.


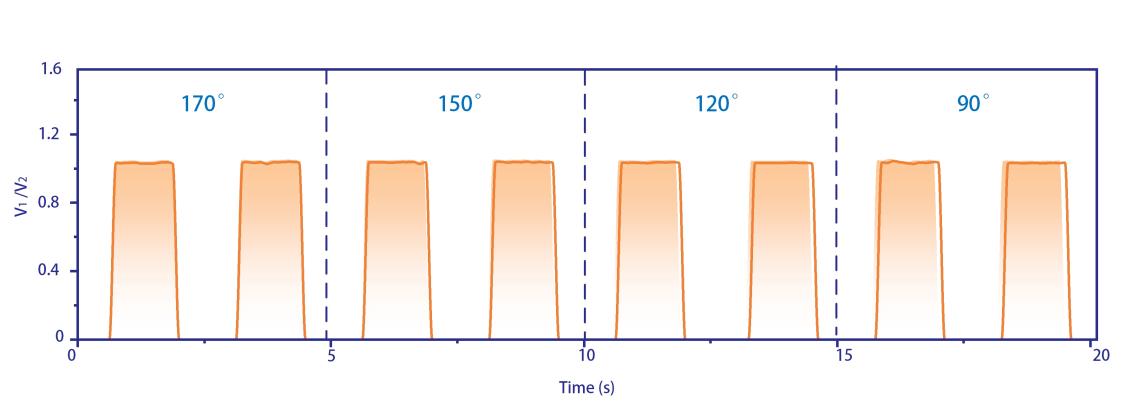


**Figure S5.** Response of NDIPD at different degrees of bending. NDIPD response at different bending angles.

In this test, the response signal of NDIPD was obtained by applying machinery to TP3 after bending the NDIPD at four degrees: 170°, 150°, 120°, and 90°. The result shows that the NDIPD can work under bending.


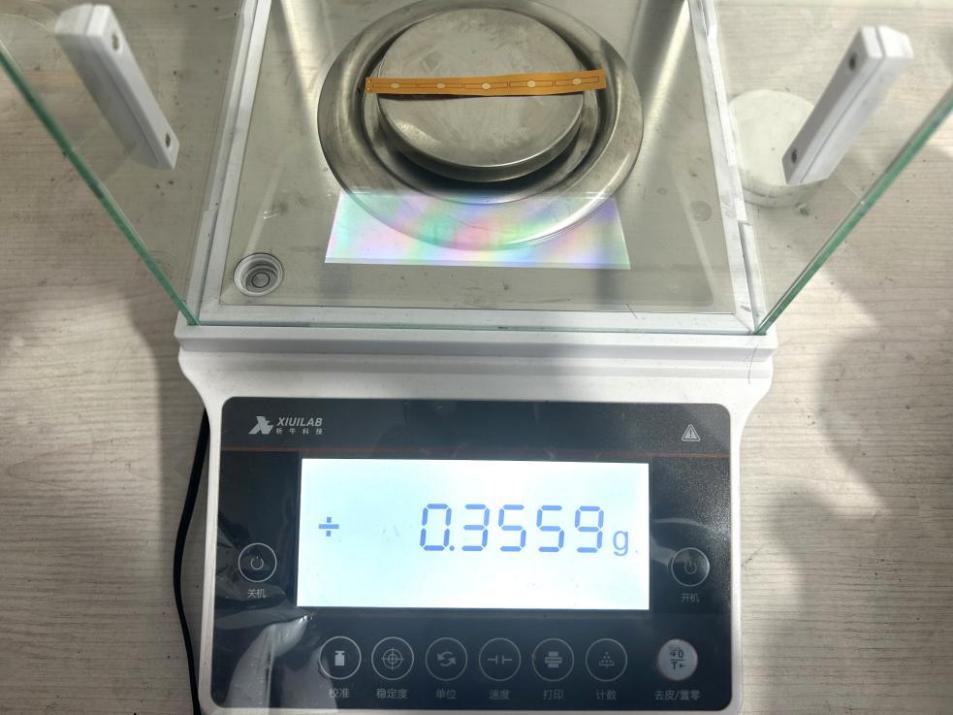


**Figure S6.** Weight of NDIPD.

The weight of the NDIPD was measured by an analytical balance and the result is 0.3559 g. This demonstrates the lightweight characteristics of the NDIPD.


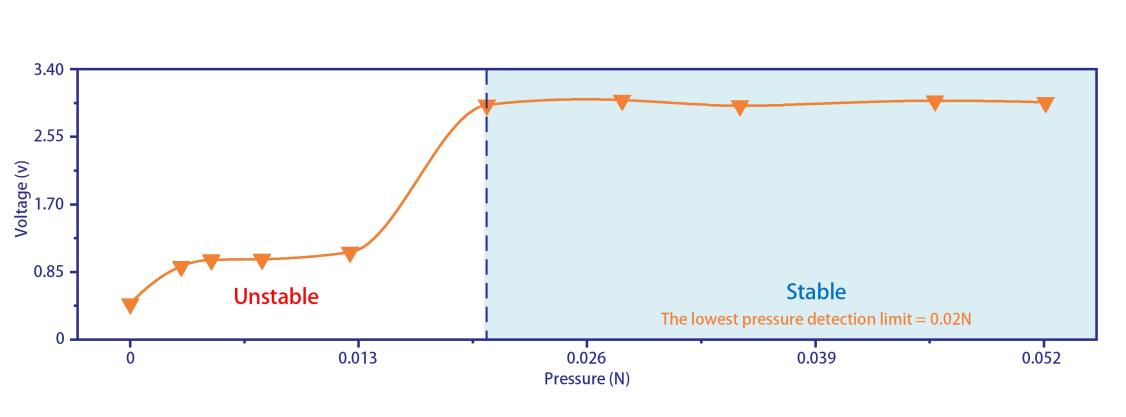


**Figure S7.** The lowest pressure detection limit of NDIPD.

The response voltage (peak-to-peak) of the NDIPD was obtained by applying mechanical force to the TP3 of the NDIPD from 0 using a push-pull force gauge and a linear sliding stage. The result shows that the lowest pressure detection limit of the NDIPD is 0.02 N.


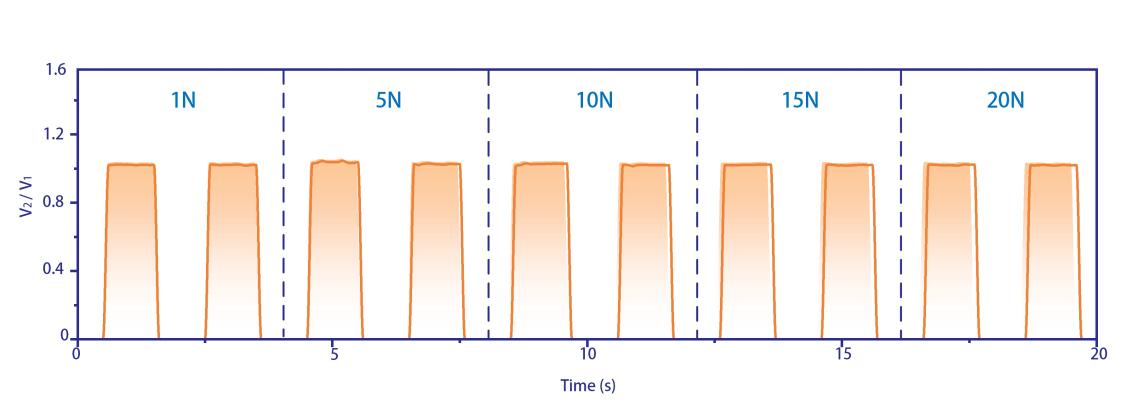


**Figure S8.** Response of NDIPD to different mechanical stimulation force levels.

In this test, five mechanical stimulations of 1 N, 5 N, 10 N, 15 N and 20 N were applied to the NDIPD using a customized actuator to obtain the peak-to-valley amplitude ratio at each point of the NDIPD. The result shows that pressure has no effect on the signal response of the NDIPD with high accuracy.

**Figure S9.** Durability of NDIPD. (a) Durability of TP2 (b) Durability of TP3 (c) Durability of TP4

The NDIPD underwent over 200,000 cyclic stimulation tests via an actuator consisting of a stepper motor, a push-pull meter, and a linear sliding stage. In this case, the contact force of NDIPD was set to 2 N, the contact frequency was set to 5 Hz, and the contact points were TP2(Figure S9a), TP3(Figure S9b) and TP4(Figure S9a). We recorded the voltage output characteristics of NDIPD during the cyclic tests. The result shows that the response signal of the NDIPD was almost unaffected even when the number of stimulations exceeded 200,000. This reflects that the NDIPD has good durability performance.


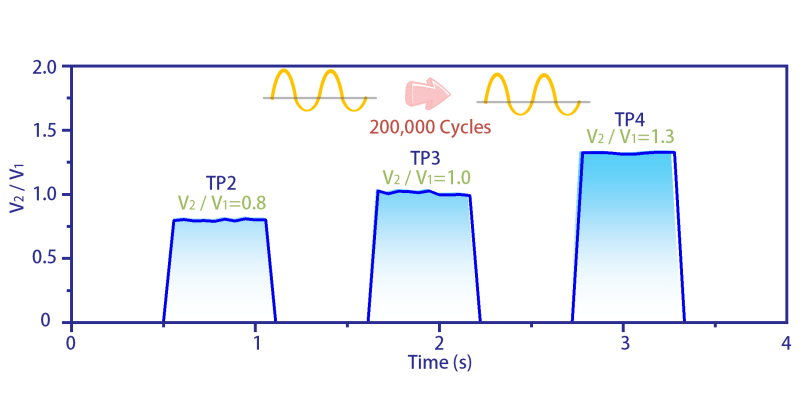


**Figure S10.** The peak-to-valley amplitude ratio of the NDIPD when TP2-TP4 is subjected to mechanical stimulation after 200,000 cycles .


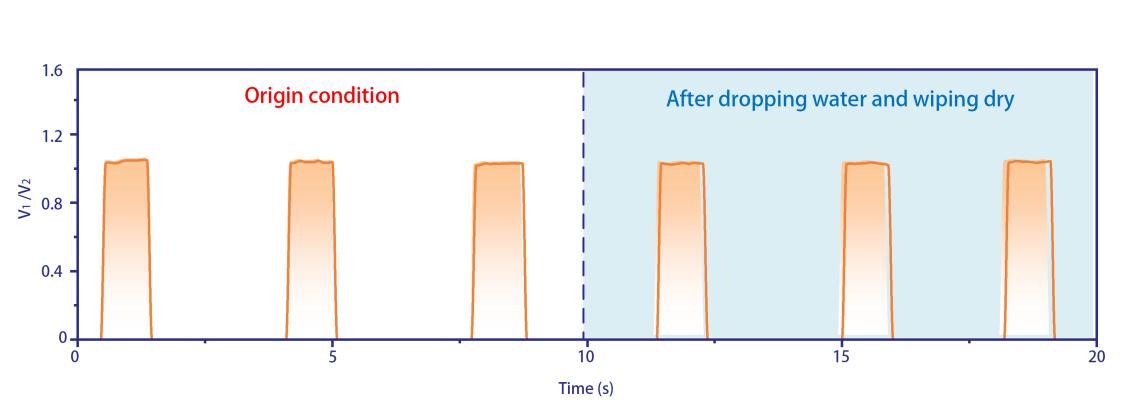


**Figure S11.** Waterproof capability of NDIPD.

The NDIPD was covered with water droplets for 5 minutes, after which the NDIPD was dried with a paper towel and mechanical simulations were applied at TP3 of the NDIPD. The result confirms the waterproof capability of NDIPD is effective.


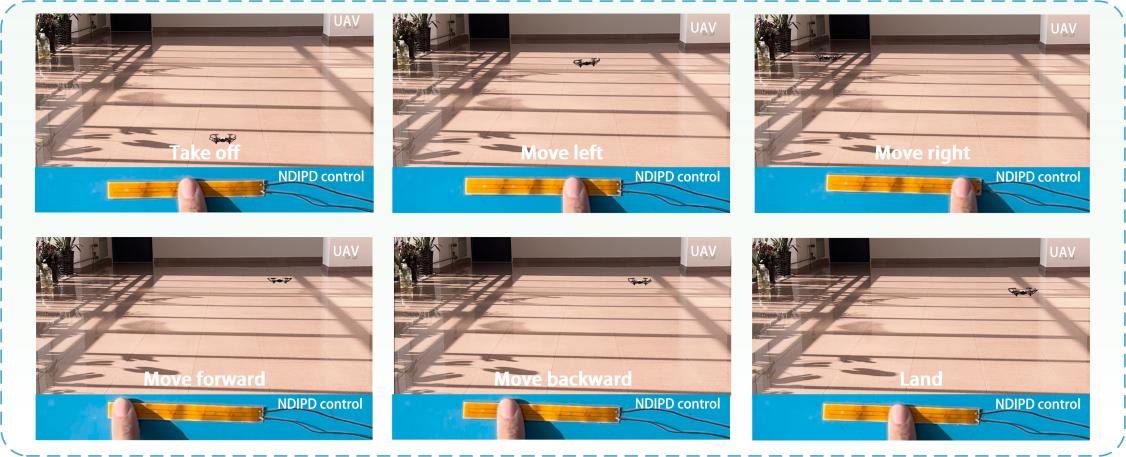


**Figure S12.** Typical photograph of an NDIPD-based UAV control platform controlling a UAV as it sequentially performs take off, move left, move right, move forward, move backward, and land.


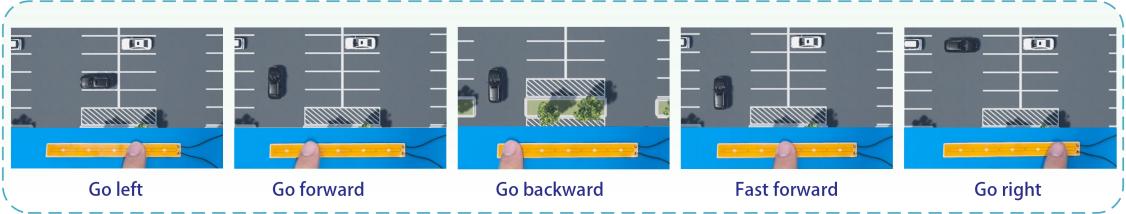


**Figure S13.** Typical photograph of an NDIPD-based virtual vehicle control platform controlling a virtual vehicle while executing go left, go forward, go backward, fast forward, and go right in sequence.


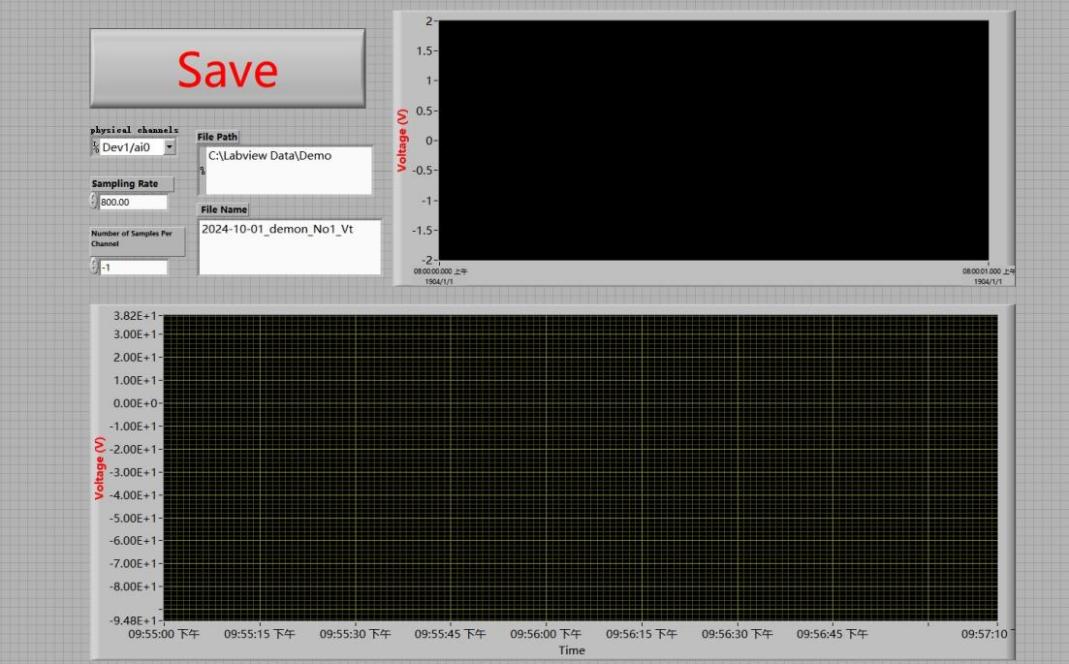


**Figure S14.** The LabVIEW signal acquisition program provides a front panel interface for displaying waveform graphs.


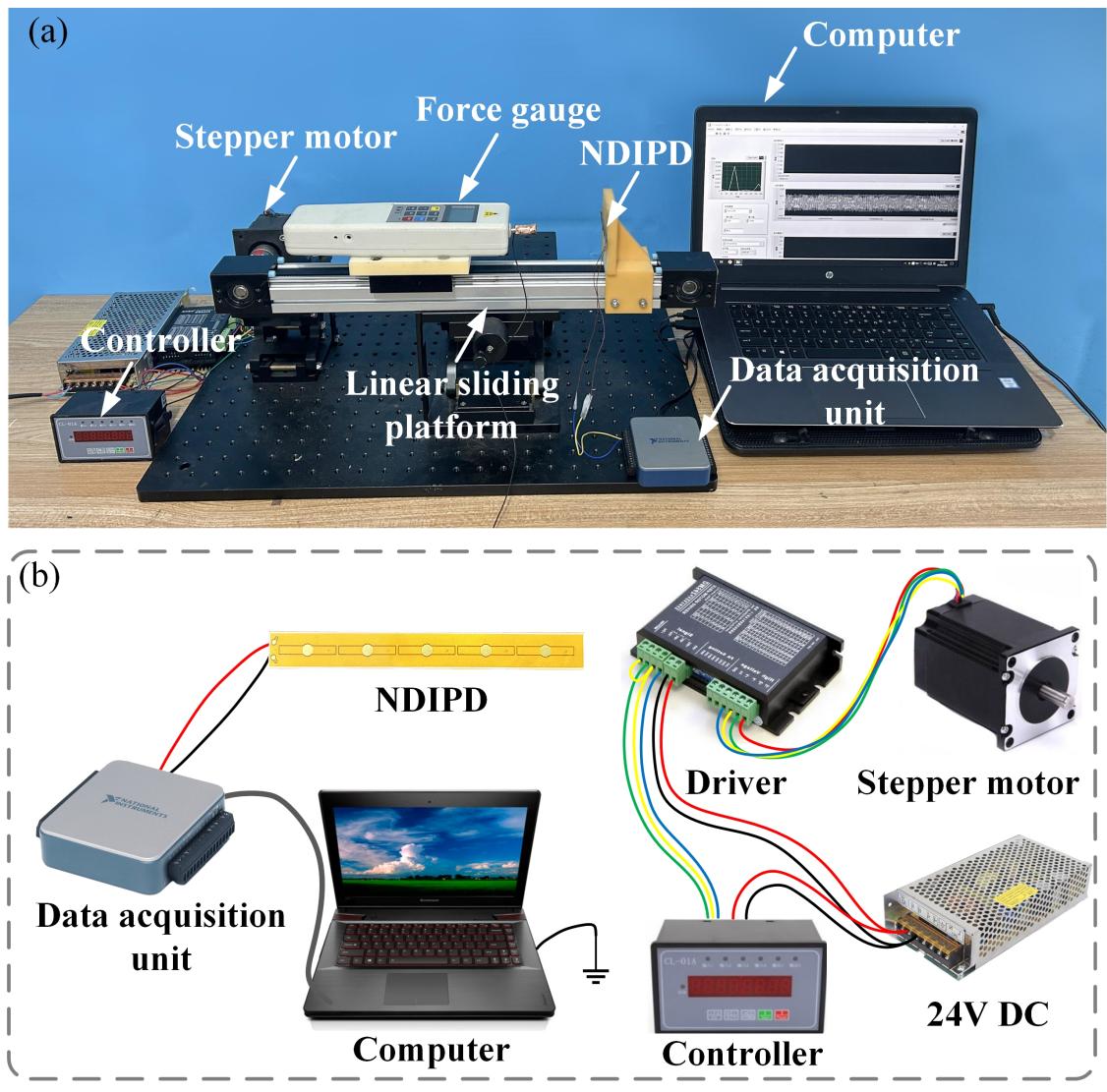


**Figure S15.** Display of experimental equipment. (a) Digital photograph of the NDIPD performance test rig. (b) Diagram of the wiring connections for NDIPD testing.

2. Supplementary Tables

**Table S1** Comparing the data from this study with those reported in the relevant literature.

| Detection limit | Environmental adaptability  (affected by the environment) | Cycle durability times | Accuracy | Response time | Self-powered | Number of interfaces | Reference |
| --- | --- | --- | --- | --- | --- | --- | --- |
| 0.02N | No | 300000 | >97% | 5ms | Yes | 2 | Ref 1  （Ref 36 in manuscript） |
| High | No | 25000 | 90% | 26.9ms | Yes | 1 | Ref 2  （Ref 54 in manuscript） |
| — | Yes | low | — | 40ms | No | 2 | Ref 3  （Ref 55 in manuscript） |
| 0.5N | No | 10000 | — | 21ms | No | 1 | Ref 4  （Ref 56 in manuscript） |
| **0.02N** | **No** | **200000** | **>97%** | **20ms** | **Yes** | **1** | **our work** |

**References**

1. K. Ba, G. Liu, G. Ma, C. Chen, L. Pu, X. He, X. Chen, Y. Wang, Q. Zhu, D. Wang, L. Liu, B. Yu, X. Kong, Z. Han, L. Ren, *Cell Rep. Phys. Sci.* **2024**, *5* (7). <https://doi.org/10.1016/j.xcrp.2024.102048.>

[2] S. Ding, D. Zhao, Y. Chen, Z. Dai, Q. Zhao, Y. Gao, J. Zhong, J. Luo, B. Zhou, *Adv. Sci.* **2024**, *11* (13), 2302782. https://doi.org/10.1002/advs.202302782.

[3] G. Gao, F. Yang, F. Zhou, J. He, W. Lu, P. Xiao, H. Yan, C. Pan, T. Chen, Z.L. Wang, *Adv. Mater.* **2020**, *32* (50), 2004290. https://doi.org/10.1002/adma.202004290.

[4] X. Liao, W. Song, X. Zhang, C. Yan, T. Li, H. Ren, C. Liu, Y. Wang, Y. Zheng, *Nat. Commun.* **2020**, *11* (1), 268. https://doi.org/10.1038/s41467-019-14214-x.

3. Supplementary Notes

**Note S1. Working Principle of NDIPD.**

The basic Working Principle of NDIPD is the combination of human body-coupling and two half-wave rectifiers. Figure S1. and Figure S2. show an equivalent circuit diagram and working principle of coupling between the power-frequency electromagnetic field, the human body, and the data acquisition unit, where *R*_NDIPD_ is the equivalent total resistance of the NDIPD, *R*_iso_ is the equivalent isolation resistance of the data acquisition unit, *C*_p_ is the coupling capacitance between the power grid and the human body, *C*_b_ is the coupling capacitance between the human body and the earth, *C*_iso_ is the ground capacitance of the data acquisition unit, and *f* is the coupled power grid frequency, *V*_a_ is the maximum voltage of the power grid, and *C*_1_ is the coupling capacitance between the power line and the external conductor of the data acquisition unit, which is negligible because it is very small. When the NDIPD is not subjected to external mechanical stimulation, the switch *S*_1_ in the equivalent circuit is in the disconnected state, and the body-coupled signal is not transmitted to the NDIPD. When the NDIPD is subjected to external mechanical stimulation, the body-coupling will provide alternating signals with symmetrical positive and negative amplitudes to the NDIPD. The resulting loading voltage can be expressed by the following equation [33]:

| $V_{\text{NDIPD}}=\frac{C_{\text{p}}R_{\text{NDIPD}}}{\left(R_{\text{NDIPD}}+R_{\text{iso}}+\frac{1}{2\pi fC_{\text{iso}}}\right)\left[C_{\text{p}}+C_{\text{b}}+\frac{C_{\text{iso}}}{2\pi f\left(R_{\text{iso}}+R_{\text{NDIPD}}\right)C_{\text{iso}}+1}\right]}\cdot V_{\text{a}}$ | (S1) |
| --- | --- |

It can be seen that the magnitude of *C*_p_, *C*_b_, and *C*_iso_ will vary in different environments, which result in the loading voltage *V*_NDIPD_ of the NDIPD varying with the environment. After the alternating signal provided by the body-coupling passes through the touch point of the NDIPD, the two branches formed on both sides of the touch point can be equated to several series-connected resistors and diodes (Figure 3a), in which this alternating signal will be split into two response signals, which will pass through different modulation circuits. One way of the signal will pass through the resistors on the left side of the touch point and the diode that is positively conducting, and will form a positive half-wave after flowing through the *R*_s_ signal. Another signal will flow through the resistor on the right side of the touch point and the negatively conducting diode, forming a negative half-wave signal after flowing through *R*_s_. The two signals are superimposed on *R*_s_ to make the signals become positive and negative asymmetric sensing signals. The resulting amplitudes are *V*_2_ and *V*_1_, respectively, and are given by the following equation:

| $V_{2}=\frac{R_{\text{s}}}{R_{\text{l}}+R_{\text{s}}}\cdot V_{\text{NDIPD}}$ | (S2) |
| --- | --- |
| $V_{1}=\frac{R_{\text{S}}}{R_{\text{total}}-R_{\text{l}}+R_{\text{S}}}\cdot V_{\text{NDIPD}}$ | (S3) |

Where *R_total_* is the total resistance of *R*_1_, *R*_2_, *R*_3_, and *R*_4_ in series and *R*_l_ is the total resistance to the left of the touch point. Combining the Equation. S2 and Equation. S3 the peak-to-valley amplitude ratio can be expressed as:

| $\frac{V_{2}}{V_{1}}=\frac{R_{\text{l}}+R_{\text{s}}}{R_{\text{total}}-R_{\text{l}}+R_{\text{s}}}$ | (S4) |
| --- | --- |

As illustrated in Equation. S4, the peak-to-valley amplitude ratio remains constant regardless of the surrounding environment. Since the total resistance *R*_total_ of *R*_1_, *R*_2_, *R*_3_, and *R*_4_ in series on the NDIPD is unchanged, the signal superposition resistance *R*_s_ is unchanged, and *R*_l_ corresponding to different positions is changed, the position of the mechanical stimulation can be obtained by measuring the peak-to-valley amplitude ratio in the response signal without interference from external factors.

**Movie S1.** Application of NDIPD in the control of the Snake game.

**Movie S2.** Application of NDIPD in the control of the UAV.

**Movie S3.** Application of NDIPD in the control of the Virtual Vehicle.
